# Seroepidemiological study of SARS-CoV-2 infection in East Java, Indonesia

**DOI:** 10.1371/journal.pone.0251234

**Published:** 2021-05-06

**Authors:** Ni Luh Ayu Megasari, Takako Utsumi, Laura Navika Yamani, Juniastuti, Emily Gunawan, Koichi Furukawa, Mitsuhiro Nishimura, Maria Inge Lusida, Yasuko Mori

**Affiliations:** 1 Indonesia-Japan Collaborative Research Center for Emerging and Re-Emerging Infectious Diseases, Institute of Tropical Disease, Airlangga University, Surabaya, Indonesia; 2 Center for Infectious Diseases, Kobe University Graduate School of Medicine, Hyogo, Japan; 3 Department of Epidemiology, Faculty of Public Health, Airlangga University, Surabaya, Indonesia; National Institute for Infectious Diseases Lazzaro Spallanzani-IRCCS, ITALY

## Abstract

Coronavirus disease 2019 (COVID-19) caused by severe acute respiratory syndrome coronavirus 2 (SARS-CoV-2) has led to a global pandemic, including Indonesia. However, there are only limited data regarding the precise prevalence of the COVID-19 pandemic in Indonesia. Here, to estimate the magnitude of SARS-CoV-2 infection in East Java, Indonesia, we investigated the prevalence of immunoglobulin G (IgG) antibodies. We enrolled 1,819 individuals from June to December 2020 and observed that the subjects’ overall prevalence of IgG antibody to SARS-CoV-2 was 11.4% (207/1,819). The prevalence of anti-SARS-CoV-2 antibodies differed significantly between the job/occupation groups (P = 0.0001). A greater prevalence of IgG was detected in laboratory technicians (who take samples from suspected cases and deal with polymerase chain reaction [PCR] procedures, 22.2%) compared to medical personnel who see and take direct care of patients with COVID-19 (e.g., physicians and nurses, 6.0%), other staff in medical facilities (2.9%), general population (12.1%) and non-COVID-19 patients (14.6%). The highest prevalence among age groups was in the 40–49-year-olds (14.8%), and the lowest prevalence was in the 20–29-year-olds (7.4%). However, the younger population still showed a higher prevalence than generally reported, suggesting greater exposure to the virus but less susceptibility to the disease. A geographical difference was also observed: a higher prevalence in Surabaya (13.1%) than in Jombang (9.9%). In conclusion, the COVID-19 outbreak among asymptomatic populations was characterized by a high prevalence of infection in East Java, Indonesia.

## Introduction

Coronavirus disease 2019 (COVID-19) is a respiratory illness caused by the newly discovered severe acute respiratory syndrome coronavirus 2 (SARS-CoV-2). This novel virus was initially identified in Wuhan, China in December 2019 and continues to plague the world [1]. Globally, over 110 million positive cases with >2.5 million deaths had been reported to the World Health Organization (WHO) as of 1 March 2021 [2]. Indonesia ranks 18th worldwide in terms of cumulative cases and 12th in terms of deaths. Although the first confirmed COVID-19 cases in Indonesia were reported on 2 March 2020, a month later than its neighboring countries, the nation’s morbidity and mortality figures have risen to become the worst among its Southeast Asian counterparts. As of 1 March 2021, 1,341,314 individuals in Indonesia had been confirmed positive, with 85.9% recovery and 2.7% case-fatality rates (https://covid19.go.id/peta-sebaran-covid19). Despite the implementation of large-scale social restrictions and "new normal" protocols in various parts of Indonesia, SARS-CoV-2 infection is steadily expanding.

Worldwide, the diagnosis of COVID-19 is based mainly on the detection of SARS-CoV-2 by real-time reverse transcription-polymerase chain reaction (RT-PCR) in a nasopharyngeal swab [3]. In Indonesia however, the precise positive rate may not have been revealed by this method, because nasopharyngeal swabs were not widely available across the country until recently. In addition, SARS-CoV-2 infection has a broad disease spectrum with clinical manifestations ranging from asymptomatic to respiratory distress [4]. Asymptomatic and mildly infected individuals may not be tested, and their infection statuses would thus remain unknown, resulting a potentially underestimated magnitude of SARS-CoV-2 infection in the population.

The majority of humans need to develop immunity against SARS-CoV-2 in order to suppress viral transmission. A seroepidemiological survey can be used to measure the proportion of individuals who have already acquired SARS-CoV-2-specific antibodies — which might protect them from subsequent infections. Seropositivity indirectly reflects the extent of SARS-CoV-2 exposure in the population, and the seropositive status may include individuals with subclinical infections. The infection rate could thus be more accurately estimated by a seroepidemiological survey, which would support crucial public health decisions during the COVID-19 pandemic [5].

Seroprevalence was particularly found to vary geographically with higher rates of seropositivity in the denser urban areas compared to rural areas [6]. The epidemiological trend also implicates SARS-CoV-2 spread among rural communities only later in the epidemic [7] which would require sound anticipatory interventions.

Many seroepidemiological surveys have examined seropositivity, focusing on households [8–11] or specific groups of people, e.g., healthcare workers [12–14], blood donors [15, 16], or dialysis patients [17, 18]. A large-scale seroepidemiological survey involving more diverse groups of people among urban and rural communities is necessary to grasp the overall picture of SARS-CoV-2 infection. We conducted the present study to evaluate the seroprevalences of SARS-CoV-2 infection in East Java, Indonesia.

## Materials and methods

### Study participants

**Fig 1:**
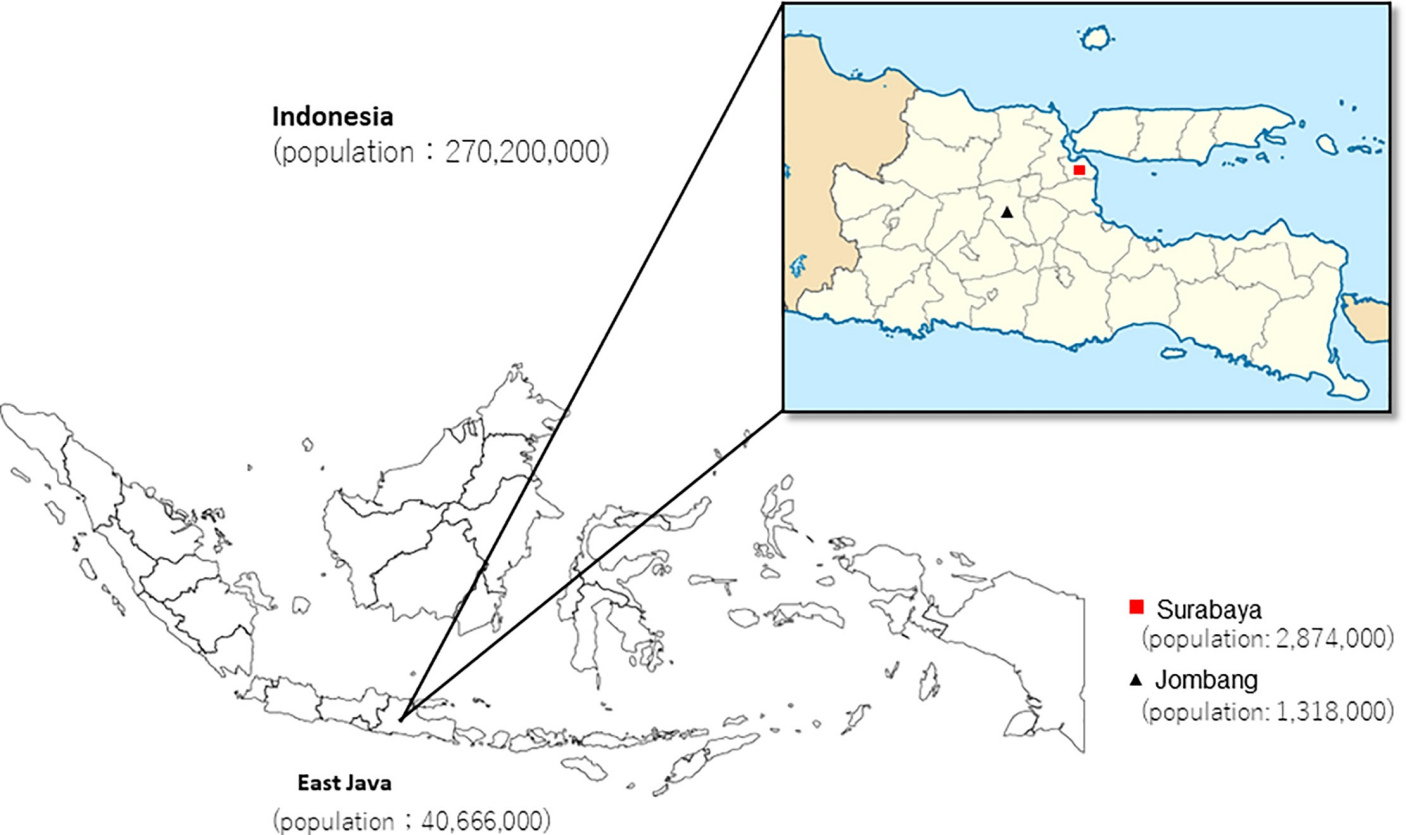
Geographic location of sample collection sites in this study. Map source: MapSVG.

From June to December 2020, 1,819 individuals over the age of 16 (mean age: 38.6 ± 14.9 years; 960 females [52.8%] and 859 males [47.2%]) were recruited from the cities of Surabaya (urban, n = 846) and Jombang (rural, n = 973), East Java, Indonesia (Fig 1) in response to an offer of free serological examinations on SARS-CoV-2. Free serological examinations were initially offered to 1,821 participants, of which 3 were excluded due to incomplete demographic data. Almost all of the participants were asymptomatic when they provided their blood samples. We classified the participants into five groups according to their occupations: (1) medical personnel who were providing direct care to patients with COVID-19; (2) laboratory technicians who had taken nasopharyngeal swab samples and/or performed the PCR procedure for the virus; (3) staff other than those in categories (1) and (2) in medical facilities; (4) general population; and (5) non-COVID-19 patients who had visited an outpatient clinic or had been hospitalized for any reason but were without COVID-19 symptoms. We also classified the participants into six age groups: 16–19, 20–29, 30–39, 40–49, 50–59, and ≥60 years. Twenty-seven samples from the patients diagnosed with COVID-19 and hospitalized in Jombang were designated as positive controls. The 27 patients were diagnosed by a polymerase chain reaction (PCR) test and clinical manifestations, which revealed 25 moderate cases, one severe case, and one critical-stage case according to the criteria of the Indonesian COVID-19 Treatment Guidelines [19]. Although pre-existing serological examination results of the 27 patients were available (as they were mandatory for COVID-19 patient hospitalization), we re-collected their blood samples and also performed free serological testing according to our research protocol to ensure reliable results.

The demographic and health characteristics of the study participants were obtained by an interviewer-administered questionnaire (S1 File). The participants reported their own ethnicities: Javanese (n = 1,611, 88.6%), Chinese (n = 92, 5.1%), and Madurese (n = 44, 2.4%) (Table 1). The sample collection sites were in Surabaya (the capital city of the province of East Java) with approx. 2,874,000 residents in 2020, and Jombang (located in the same province) with an estimated 2020 population of 1,318,000 [20]. This study was approved by the Institutional Ethics Committee of Kobe University Graduate School of Medicine, Kobe, Japan (approval no. B200600) and the Ethics and Law Committee of Airlangga University Hospital, Surabaya, Indonesia (Ethical approval no. 163/KEH/2020). Written informed consent was obtained from all study participants prior to sample collection.

**Table 1:** Demographic characteristics and IgG positivity of the cohort.

Characteristic	Total	IgG-positive	IgG-negative	P-value
	n = 1,819	n = 207	n = 1,612	
Sex, n (%):				
Female	960 (52.8)	115 (12.0)	845 (88.0)	
Male	859 (47.2)	92 (10.7)	767 (89.3)	0.395
Age groups, yrs, n (%):				
16–19	69 (3.8)	8 (11.6)	61 (88.4)	
20–29	598 (32.9)	44 (7.4)	554 (92.6)	
30–39	366 (20.1)	51 (13.9)	315 (86.1)	
40–49	271 (14.9)	40 (14.8)	231 (85.2)	
50–59	345 (19.0)	42 (12.2)	303 (87.8)	
≥60	170 (9.3)	22 (12.9)	148 (87.1)	0.008
Occupation, n (%):				
Medical personnel	167 (9.2)	10 (6.0)	157 (94.0)	
Laboratory technician	18 (1.0)	4 (22.2)	14 (77.8)	
Other staff in medical facility	140 (7.7)	4 (2.9)	136 (97.1)	
General population	1,159 (63.7)	140 (12.1)	1019 (87.9)	
Non-COVID-19 patient	335 (18.4)	49 (14.6)	286 (85.4)	0.0001
Sampling place, n (%):				
Surabaya	846 (46.5)	111 (13.1)	735 (86.9)	
Jombang	973 (53.5)	96 (9.9)	877 (90.1)	0.029
Ethnicity, n (%):				
Java	1,611 (88.6)	186 (11.5)	1,425 (88.5)	
Chinese	92 (5.1)	10 (10.9)	82 (89.1)	
Madurese	44 (2.4)	3 (6.8)	41(93.2)	
Others	72 (3.9)	8 (11.1)	64 (88.9)	0.558

### Sample collection

Blood samples were collected at two communities and one medical research center in Surabaya and one private hospital in Jombang. The inclusion criteria were workers (medical personnel, laboratory technician and other staff) and non-COVID-19 patients in medical facilities as well as general population in the community. The exclusion criterion was persons less than 16 years old. Five ml of peripheral blood was collected from each participant and then transported to the Institute of Tropical Disease, Airlangga University. The samples were left at room temperature for 30 min to allow clotting, then centrifuged at 1,300 relative centrifugal force (RCF) for 10 min in a swinging bucket rotor. The serum was then separated and transferred into two individual tubes, and frozen at −80°C until further use.

### Qualitative detection of immunoglobulin G (IgG) antibody to SARS-CoV-2

IgG antibodies for SARS-CoV-2 in each serum sample were analyzed by the antigen colloidal gold of the 2019-nCoV IgG detection kit (Nanjing Vazyme Medical Technology, Nanjing, China). The manufacturer-reported value of the kit’s sensitivity is 91.54%, and its specificity is 97.02%.

### Statistical analysis

The statistical analysis was performed by the chi-squared test for categorical variables using SPSS Statistics 17.0 (Advanced Analytics, Tokyo). Results were considered significant at P<0.05.

## Results

### Prevalence of anti-SARS-CoV-2 antibodies

The overall prevalence of anti-SARS-CoV-2-IgG was 11.4% (207/1,819). Table 1 provides the prevalence data of anti-SARS-CoV-2-IgG in the different demographic settings. The comparison of prevalence by sampling location revealed that the prevalence of anti-SARS-CoV-2-IgG was significantly higher in Surabaya (13.1%; 111/846) compared to Jombang (9.9%; 96/973) (P = 0.029). It should be noted that there was also a significant difference in the prevalence of anti-SARS-CoV-2 antibodies among the occupation groups (P = 0.0001). The occupation group with the highest prevalence was the laboratory technicians (22.2%) followed by the non-COVID-19 patients (14.6%), general population (12.1%), medical personnel (6.0%), and other medical staff (2.9%).

The prevalence of anti-SARS-CoV-2 antibodies also differed significantly among the age groups (P = 0.008). The of 40–49 age group had the highest prevalence (14.8%), and the 20–29 age group showed the lowest prevalence (7.4%). There were no significant differences in anti-SARS-CoV-2 antibodies in terms of sex or ethnicity.

### Gender distribution of positive IgG antibodies stratified by age group

**Fig 2:**
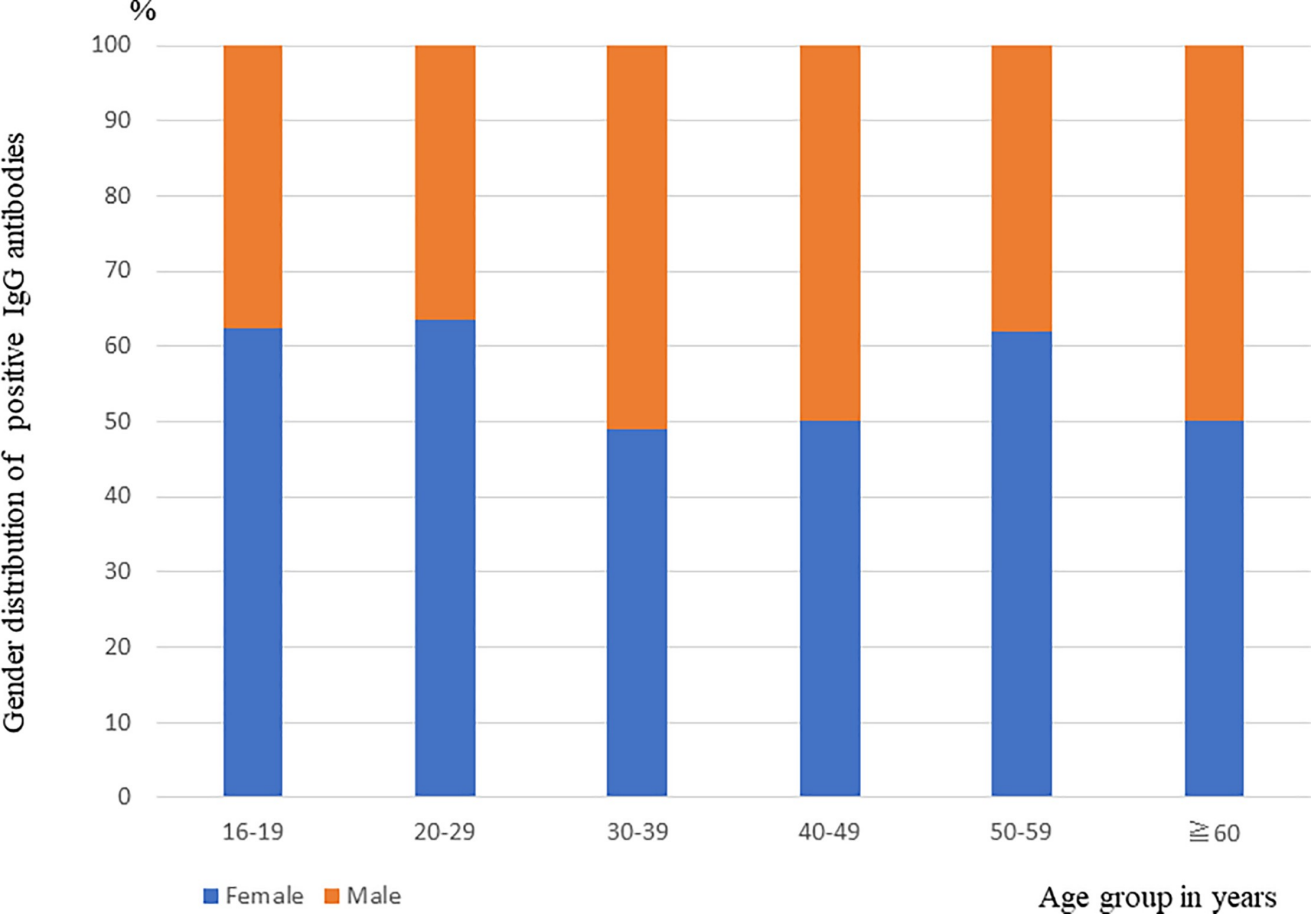
Age distribution of IgG-positive cases stratified by sex: East Java, Indonesia (n = 207 cases).

A total of 207 individuals tested positive for IgG antibodies, of which 115 were female (Table 1). The distributions of positive IgG antibodies in the females in the age groups 16–19, 20–29 and 50–59 years were much higher than those in the males, although the differences were not significant (Fig 2).

### Prevalence of anti-SARS-CoV-2 antibodies in the control group

Sera from patients with COVID-19 in the hospital were used as positive controls and showed the IgG antibody positive rate of 92.6% (25/27).

## Discussion

The COVID-19 pandemic caused by SARS-CoV-2 is ongoing in Indonesia as in many other countries as of March 2021. The numbers of reported laboratory-confirmed cases of SARS-CoV-2 infection have been gradually decreasing, although Indonesia continues to have the worst in Southeast Asia. The results of our present seroepidemiological study focusing on the seroprevalence of SARS-CoV-2 IgG antibodies in an asymptomatic population revealed significant differences in the prevalence of IgG antibodies by occupational category, age group, and sample collection sites. Almost all sites on Java Island including our sample collection sites Surabaya and Jombang have presented clusters where many people gather such as schools, dormitories, markets, etc. Our study cohort was considered to be from pandemic areas and to be representative of a subclinical condition.

The overall positive rate of IgG antibodies against SARS-CoV-2 in this study was 11.4%, suggesting a higher positivity among this asymptomatic population compared to 8.3% in an asymptomatic population in Brazil [21] and 1.2% of asymptomatic outpatients in Germany [22]. However, higher prevalences have been reported in asymptomatic populations, e.g., Veracruz (Southeastern Mexico) at 21.3% [23] and Japan at 17.9% [24].

On the other hand, among the patients with COVID-19 in this study (the control group), the positive rate of IgG antibodies was 92.6% (25/27). The two patients with non-reactive IgG results were at the earlier stage of illness according to their report of the onset of illness. It is likely that these patients still lacked the IgG antibodies, since it usually appears within 14 days after infection.

The seroprevalence of IgG antibodies against SARS-CoV-2 varies according to different populations [25], and medical personnel, elderly people, and those with underlying health conditions are at high risk [26]. In our cohort, the prevalence of IgG antibodies was highest in the laboratory technician (22.2%), suggesting that those who take the samples from suspected cases or who deal with the samples for PCR testing are at higher risk than physicians and nurses who directly see or care for patients with COVID-19. These findings are consistent with a study conducted in India [25]. However, data that concern the higher risk of infection in laboratory technicians as a separate category from other medical staff are still rare. Nasopharyngeal swab sampling itself is considered an aerosol-generating procedure which may pose higher SARS-CoV-2 infective risk towards the laboratory technicians who collected large quantities of swabs daily. The use of personal protective equipment (PPE) by medical staff including laboratory technicians is mandatory in Indonesia, and we could thus expect that laboratory technicians would be equally at high risk of SARS-CoV-2 infection. However, our analyses demonstrated that the infection risk among medical personnel who see and take direct care of COVID-19 patients and the administrative staff at medical facilities was much lower than those of the other occupational groups in this study. Similar studies focusing on medical personnel from other countries/areas reported rates as low as 0% in Japan [27] and Malaysia [28] and as high as 13.7% in New York City [12]. Although the use of PPE is an effective method to avoid infectious diseases [27, 28], there are multiple factors that predispose any given individual to infection. One possibility that may involve the present finding in laboratory technicians is viral transmission outside of the medical facilities, e.g., in living areas. Several studies have reported a high proportion of community-acquired SARS-CoV-2 infections among healthcare workers [29] as well as traceable positive contacts within their households sharing identical RNA sequences [30, 31].

A geographic difference in the prevalence of IgG antibodies was observed in this study, i.e., a higher prevalence in Surabaya compared to Jombang. This finding is again directly proportional to the higher COVID-19 incidence of 612 cases per 100,000 (https://lawancovid-19.surabaya.go.id/) in Surabaya compared to 142 cases per 100,000 in Jombang at the end of our study period. These data suggest a transmission pattern from the provincial capital to surrounding areas as described [32].

Our results also demonstrated that COVID-19 infection was widely present in all age groups, although among the age groups, the positive rate of IgG antibodies in the participants >30 years old was significantly higher than that in the younger population, similar to another report [9]. However, the young participants in our study showed a higher prevalence than generally reported, suggesting greater exposure to the virus but less susceptibility to the disease [11]. With regard to sex, we observed no significant difference between the males and females, as in another study [18]. The same is true of the three ethnic groups examined herein.

In conclusion, the COVID-19 outbreak among asymptomatic populations in East Java, Indonesia were characterized by a high prevalence of infection. Laboratory technicians were at the highest risk. A mass vaccination program was begun in January 2021 in Indonesia as in some other countries, and it is crucial to conduct continuous seroloepidemiological surveys to understand the infection dynamics and the achievement of herd immunity.

## Supporting information

S1 File Survey questionnaires (Indonesian version and English version). (PDF)
