# Supplementary material for: Seroepidemiological study of SARS-CoV-2 infection in East Java, Indonesia
Source: PLoS One. 2021 May 6;16(5):e0251234. doi: 10.1371/journal.pone.0251234 (PMC8101714; doi:10.1371/journal.pone.0251234)
Supplement: S1 File — (PDF) [file pone.0251234.s001.pdf]

KUESIONER

Tanggal (/   /   )

1. Nomor ID :
2. Nama subjek :
3. Jenis kelamin : ☐ Pria ☐ Wanita
4. Suku : ☐ Jawa ☐ Madura ☐ Lainnya (   )
5. Umur : (   ) tahun
6. Pekerjaan : ☐ Tenaga Medis (Dokter/Perawat)

   ☐ Pegawai Rumah Sakit (selain dokter dan perawat)

   ☐ Pasien (☐Dengan COVID-19, ☐Non-COVID-19, ☐Tidak diketahui)

   ☐ Staff laboratorium

   ☐ Karyawan/Pegawai Kantor

   ☐ Lainnya (   )
7. Tempat tinggal

   ☐ Jawa Timur (Nama Daerah:)

   ☐ Luar Jawa Timur (Nama Daerah:)

   Jika subjek adalah pasien COVID-19,

   ☐ Waktu munculnya gejala (tanggal,   bulan,   tahun)

   ☐ Waktu masuk rumah sakit (tanggal,   bulan,   tahun)

   Penyakit penyerta; ☐ Ada (   ), ☐ Tidak ada

   Hasil PCR: ☐ Positif ☐ Negatif

8. Gejala saat ini:

   ☐ Tidak ada ☐ Ada

   Jika "ada" gejala;

   ☐ Demam (℃), ☐ batuk, ☐ sakit tenggorokan,

   ☐ pilek/hidung tersumbat, ☐ letih, ☐ sesak nafas,

   ☐ pneumonia, ☐ lainnya (   )

9. Riwayat perjalanan ke luar negeri atau luar kota (dalam negeri) sejak Desember 2019 hingga sekarang:

   ☐ Tidak ada ☐ Ada

   Jika "ada" riwayat perjalanan;

   Negara/kota yang dikunjungi :

   Waktu dan lama kunjungan :

10. Riwayat kunjungan ke rumah sakit, puskesmas, klinik, atau fasilitas kesehatan lainnya sejak Desember 2019:

☐ Tidak

☐ Ya

Jika "ya";

Nama fasilitas kesehatan :

Frekuensi, waktu, dan durasi :

Tujuan :

11. Riwayat kontak langsung dengan orang yang suspek ataupun positif COVID-19:

☐ Tidak

☐ Ya

Jika "ya";

Waktu, frekuensi, dan durasi terjadinya kontak :

12. Riwayat *swab test* COVID-19:

☐ Tidak

☐ Ya

Jika "ya";

Frekuensi pemeriksaan :

Waktu pemeriksaan :

Hasil :

Alasan / Indikasi :

Gejala :

☐ Tidak ada

☐ Ada

Jika "ada" gejala;

☐ Demam (℃),	☐ batuk,	☐ sakit tenggorokan,
☐ pilek/hidung tersumbat,	☐ letih,	☐ sesak nafas,
☐ pneumonia,	☐ lainnya ( )	

13. Riwayat rawat inap di rumah sakit akibat COVID-19:

☐ Tidak

☐ Ya

Jika "ya";

Nama fasilitas kesehatan :

Waktu dan durasi :

QUESTIONNAIRE

Date (/   /   )

1. ID :
2. Name :
3. Sex : ☐ Male ☐ Female
4. Ethnicity : ☐ Javanese ☐ Madurese ☐ Others (   )
5. Age : (   ) years old
6. Occupation : ☐ Medical staff (Doctor/Nurse)
   ☐ Hospital staff (other than doctor and nurse)
   ☐ Patient (☐With COVID-19, ☐Non-COVID-19, ☐Unknown)
   ☐ Laboratory staff
   ☐ Office worker/staff
   ☐ Others (   )
7. Residence
   ☐ East Java (City Name:)
   ☐ Outside East Java (City Name:)
   If subject is a COVID-19 patient,
   ☐ Symptom onset (date,   month,   year)
   ☐ Hospitalization (date,   month,   year)
   Comorbidity; ☐ Yes (   ), ☐ No
   PCR Result: ☐ Positive ☐ Negative

8. Current symptoms:
   ☐ No ☐ Yes
   If “yes” for symptoms;
   ☐ Fever (℃), ☐ cough, ☐ sore throat,
   ☐ runny/congested nose, ☐ fatigue, ☐ shortness of breath,
   ☐ pneumonia, ☐ others (   )

9. History of travelling to another country or another city (domestically) since December 2019 until now:
   ☐ No ☐ Yes
   If “yes” for travel history;
   Country/city visited :
   Time and duration of visit :

10. History of visiting hospitals, primary health centers, clinics or other health facilities since December 2019:

☐ No

☐ Yes

If "yes";

Name of health facility :

Frequency, time and duration :

Purpose :

11. History of direct contact with suspected or COVID-19 cases:

☐ No

☐ Yes

If "yes";

Time, frequency and duration of contact :

12. History of COVID-19 *swab test*:

☐ No

☐ Yes

If "yes";

Examination frequency :

Time of examination :

Result :

Indication :

Symptoms :

☐ No

☐ Yes

If "yes" for symptoms;

☐ Fever (℃),	☐ cough,	☐ sore throat,
☐ runny/congested nose,	☐ fatigue,	☐ shortness of breath,
☐ pneumonia,	☐ others (	)

13. History of hospitalization due to COVID-19:

☐ No

☐ Yes

If "yes";

Name of health facility :

Time and duration :
